# Swimming-induced changes in pulmonary function:special observations for clinical testing

**DOI:** 10.1186/s13102-021-00277-1

**Published:** 2021-05-20

**Authors:** Marja Pivinen, Kari Keskinen, Heikki Tikkanen

**Affiliations:** 1 University of Turku, Turku, Finland; 2 University of Helsinki, Helsinki, Finland; 3 University of Jyvskyl, Jyvskyl, Finland; 4 University of Eastern, Eastern Finland, Finland

**Keywords:** Pulmonary function, Spirometry, Exercise, Swimming

## Abstract

**Background:**

A special improvement in pulmonary function is found in swimmers. In clinical testing the airway reactivity is observed at certain exercise intensity and target ventilation. However, in highly trained swimmers exercising in water the reactions may not function the same way. The aim was to study the combined effects of the water environment and swimming on pulmonary function and the associations with perceived symptoms.

**Methods:**

First, 412 competitive swimmers completed questionnaires concerning respiratory symptoms at different swimming intensities. Then, pulmonary function testing was performed in 14 healthy elite swimmers. Spirometry and maximal voluntary ventilation (MVV) were measured on land and in water before and after swimming. While swimming, minute ventilation (VE) tidal volume (VT) and breathing frequency (fb) were measured during competition speed swimming.

**Results:**

Swimmers reported the most symptoms at competition speed intensity swimming. In the transition from the land into the water swimming body position, the ratio of forced expiratory volume in one second (FEV_1_) and forced expiratory capacity (FVC) (FEV_1_/FVC) decreased by a mean (SD) 5.3% (3) in females and by 2.2% (5) in males. During competition speed intensity swimming, the minute ventilation (VE) had a mean of 72 and 75% of calculated maximal voluntary ventilation (cMVV) in females and in males, respectively.

**Conclusions:**

Spirometry showed sex differences in water compared to land measurements. These differences should be considered when the effects of swimming are observed. During the intensity that triggered the symptoms the most, the VE was approximately 20% higher than the target ventilations for clinical testing. These findings encourages specific modifications of clinical testing protocols for elite swimmers.

## Introduction

Special improvements in pulmonary function are reported in competitive swimmers by higher than predicted spirometry findings[1,3]. The causes for the adaptation have been explained by the special effects of swimming training and the water environment. During swimming the breathing pattern is different from the free breathing during land-based sports. While swimming, the inspiratory phase of the breathing cycle is timed to coincide with arm strokes, which limit the duration of inhalation. Therefore, to provide adequate airflow to the lungs during swimming, the special dynamics of breathing occur by a rapid inhalation near total lung capacity (TLC), negative pressure during inhalation and prolonged exhalation into water due to the necessity to coordinate breathing with the upper limb cycle [4, 5].

During immersion, the hydrostatic pressure of water causes compression into the chest wall, which affects the volume of breathing by an approximately 611% decrease in vital capacity[6]. The pressure in lungs in the prone swimming body position near the water surface is approximately 89kPa in a depth of approximately 20cm H_2_O. The hydrostatic pressure causes effects on circulation and redistribution of blood volumes in the body. The conductivity of temperature is 25 times higher in water than in air. The body reacts on by altering its heart rate by bradycardia. During maximal exercise the heart rate is reported to be 10 beats lower in water compared to the exercise on land[4]. Face immersion also causes special effects on breathing in comparison to normal breathing on land.

The swimming environment has beneficial effects on pulmonary health[7,9]. Warm and humid air relieves perceived respiratory symptoms. However, respiratory symptoms and asthma are more common among swimmers than in the general population[10, 11].

Clinical pulmonary function testing, in regard to reported respiratory symptoms and pulmonary disease in swimmers, is typically executed with a 60% ventilation level of calculated maximal voluntary ventilation [12]. This is without considering the possible effects of a water environment and target-oriented competitive swim training on lung function. Thus, in competitive swimmers, the effects of water immersion, while swimming, on pulmonary dynamics remains unclear. Therefore, the aims of this study were to determine the effects of water immersion and high-intensity competition speed swimming on pulmonary function in trained elite swimmers and also to elucidate further the water-related effects on pulmonary function and respiratory symptoms while swimming.

## Methods

First, the participants of National Swimming Championships, consisting of 412 competitive swimmers with 218 females and 194 males, having a mean of (SD) age of 18 (3), and a mean of 9 years of a competitive swimming training background were studied. Swimmers had to qualify for national championships by performing at certain level at official competitions. The qualification time for each swimming event was set according to swimming result statistics referring FINA points approximately on the level of 500. Swimmers filled in questionnaires concerning their medical background, training history and reported respiratory symptoms while swimming at different intensities. A survey was used to determine the swimming intensity, at which the competitive swimmers reported respiratory symptoms the most. The studied swimmers were well familiar with the different training intensities concerning the reporting of respiratory symptoms being cough, wheezing of breath, mucous production from airways and shortness of breath. Five different swimming intensities were described previously in a study by Pivinen et al. (2010). The intensity zone I was light endurance swimming referring to RPE 911; zone II was moderate endurance exercise between aerobic and anaerobic thresholds referring to RPE 1213; zone III was hard intensity a swimming pace between the anaerobic threshold and the minimal velocity of maximal oxygen uptake referring to vigorous RPE 1416; zone IV very hard intensity with velocity higher than zone III, with a competition specific race-pace maximizing lactic acid production referring to vigorous to near maximal effort with RPE 1718; and zone V intensity was all out maximal short sprinting referring to RPE 18.

Second, fourteen healthy elite competitive swimmers being 7 females and 7 males were studied with pulmonary function testing. The selected studied swimmers were healthy and symptom-free. They had a similar training background of 9 years and a fitness level as finalists in national championships with FINA points approximately at the level of 650850. They were also approximately at the same age with a mean age of 18 (2) years. Female swimmers had a mean (SD) height of 168 (5) cm and weighed at a mean of 62 (5) kg. Male swimmers had a height of 184 (4) cm and weighed 75 (5) kg. The BMI in both females and males was 22. This selection was set to avoid the disturbance of age, pulmonary disease, fitness levels and training background on the pulmonary function results [3, 13, 14]. All studied swimmers were non-smokers.

Pulmonary function testing in connection to a swimming environment was performed by spirometry first on land in a sitting position and then in water with a prone swimming body position before the swim. Spirometry was repeated after competition speed swimming first in water and then on land.

Spirometry testing measures the pulmonary flows and capacities, which are forced expiratory volume (FVC), forced expiratory volume in one second (FEV_1_), the ratio of FEV_1_ and FVC (FEV_1_/FVC) and peak expiratory flow (PEF). The spirometry testing was executed by the ATS and ERS guidelines [15, 16].

Maximal voluntary ventilation was determined by direct measurement (MVV) for 10s and by calculation (cMVV) according to the equation of Wasserman[17]as 35xFEV_1_.

During swimming testing, the minute ventilation (VE) was detected by the breath-by-breath method. Tidal volume (VT) and breathing frequency (fb) were also obtained. The swimming intensity for the testing was set at swimming intensity zone IV, which is the competition speed intensity according to the results of the survey. Zone IV refers to the RPE 1718 and also can be described as the areas of the highest part of the vigorous intensity and near to the maximum intensity (ACSM Guidelines of exercise testing p.146)[18].

Special equipment for water testing was used. Measurements were performed with a portable Cosmed K^4^b^2^ gas analyser. A special mouthpiece and snorkel suitable for testing in water were used in all measurements[19]. Dead space formed by the equipment was 710 ml, which consisted of the mouthpiece, 40 ml; a connecting piece, 190 ml and an expiration tube, 480 ml. The snorkel was attached to a two-way breathing valve, and the analyzer was connected to a computer with a cable in the validated testing system by Keskinen et al. (2003). This was required to be able to run the Cosmed spirometry testing program. Testing was not possible to execute wirelessly, because measuring spirometry required the cable connection. Special delay calibration with the flow calibration was performed. To ensure that the results of the measurements were comparable, the same equipment was used in all measurements.

The testing environment was at ambient temperature in the swimming hall at 3032 degrees Celsius, and air humidity was 5055%. The airborne trichloroamine level in the swimming hall was less than 0,1mg/m^3^, which followed European Union regulations[20]. The pool water temperature was 27 Celsius.

### Statistical calculations

The reproducibility of the testing methods is 35%. In this study, when two methods were compared, the difference of >10% was considered statistically significant. When the minute ventilation was estimated at 150lmin^1^ and measured or calculated at MVV 200lmin^1^[3], on the level of a<0.05 and with the number of 14 study subjects, participating in pulmonary function testing, the statistical power of 80% could be reached [21].

Risk factors for reported respiratory symptoms were calculated with odds ratios (OR). The OR between different variables that might have influenced on the prevalence of respiratory symptoms was calculated by multiple logistic regression analysis (Table 2). Further details of the method are presented by Pivinen et al. [11]. Multiple logistic regression is a technique for analyzing problems in which there are one or more independent variables that determine outcome values. Outcome is a dichotomous variable, that is, it has only two values. In this study, those outcomes are reported by symptoms and no symptoms. OR is related to the outcome values. OR greater than 1 are related to higher probability compared to a reference class and OR less than 1 are related to lower probability compared to the preference class.

A T-test was used for comparing the results between female and male swimmers. First, the normality of data was studied with a Shapiro-Wilks test. If it failed, the rank-sum Mann-Whitney test was performed. All comparisons were performed with SigmaPlot software 11.0. (Systat Software inc. San Jose, CA, USA).

Normal distribution was calculated for the swimming-induced changes on FEV_1_ both in the changes obtained by land measurements and by water measurements. Probability Mass Function was plotted for FEV_1_ of both land and water measurements changes using the NORMDIST-function in Excel.

The ethical considerations for the questionnaire and pulmonary function testing on land were evaluated and approved by the ethics committees of the Helsinki and Uusimaa hospital districts and by the University of Jyvskyl. Each test subject received information about the testing procedure and signed written consent for voluntarily participating in the testing. The procedure of the pulmonary function testing is shown in the Table 1.

**Table 1:** Pulmonary function testing procedure and timing of the testing execution

Timing	Action
1h 20min before testing	Preparation of testing equipment. Switch on the K4b2 analyzer
30min before testing	System calibration, flow calibration, special delay calibration
15min before testing	Study subject arrives ready to swim with swimming gear on, Explaining the testing procedure to the subject Filling preliminary information on testing program
Starting testing	Spirometry testing on land, study subject in sitting body position
2.30min after starting testing	Maximal voluntary ventilation testing on land and immediately transition into water prone swimming body position in water
5min after starting testing	Spirometry testing in water. The study subject float in prone swimming body position. The swimmer has a handgrip on the side of pool deck and keeps the face immersed. The swimmer wears a special swimming nose-clip and keeps a pull-buoy between tights to maintain prone swimming body position and prevent feet from sinking
7.30min after starting testing	Maximal voluntary ventilation testing in water
10min	Swimming, study subject performing programmed swimming exercise, which included 100m freestyle at competition speed intensity in the end. Ventilation during the test swimming was measured with breath-by-breath method.
0min after swimming	Spirometry testing in water environment the same way as before swimming
2.30min after swimming	Maximal voluntary ventilation testing in a water the same way as before swimming
5min after swimming	Spirometry testing on land, study subject in sitting body position the same way as before
7.30min after swimming	Maximal voluntary ventilation testing on land
10min after swimming	Finishing measurements and removing the testing equipment

## Results

### Reported symptoms and swimming intensity

The prevalence of reported respiratory symptoms among 412 competitive swimmers, who qualified for the National Championships, was 46%. The prevalence of physician-diagnosed asthma was 20%, physician-diagnosed allergy was 35%, family history of asthma was 18% and family history of allergy was 28% in competitive swimmers.

**Fig. 1:**
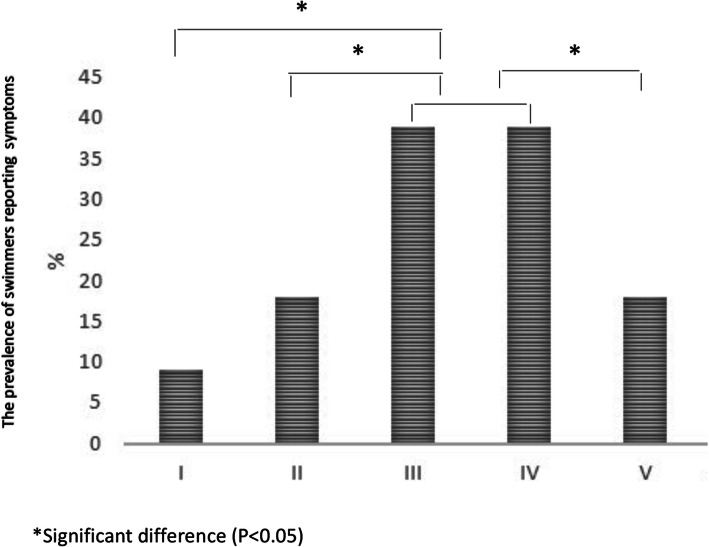
Prevalence of reported symptoms in 412 Finnish competitive swimmers at fiveswimming training intensity zones: I-V. Differences between intensities were calculatedusing multiple logistic regression analysis

When the relationship between the prevalence of reported respiratory symptoms was related to the physical strain of the swimming exercise, it was found that the swimmers reported most symptoms while swimming at the hard, zone III, and very hard, competition speed zone IV, intensities as shown in Fig.1. Competitive swimmers reported less symptoms during light (zone I), moderate (zone II) and short maximal sprinting (zone V) intensity swimming.

#### Effect of water immersion on pulmonary function

Spirometry testing on land and in water showed that the water immersion caused effects on pulmonary function findings. The transition from a land-oriented, vertical sitting body position into a water-oriented, prone swimming body position caused a 18% decrease in spirometry variables (Table 2). The swimming environment caused an observable effect on pulmonary function. This effect seems to be highly different between females and males especially in forced vital capacity (FVC) and the ratio of forced expiratory volume in one second (FEV_1_) and forced vital capacity (FVC); (FEV_1_/FVC).

**Table 2:** Effect of transition from land into a prone swimming body position in water onspirometry findings in 14 elite competitive swimmers. The effect is calculated as percent ofthe measured change out of the initial value measured on land

	Female swimmers *N*=7	Male swimmers *N*=7	*p*-value
FEV_1_ decrease in transition into water %	6.3 (3)	7.7 (3)	0.42
FVC decrease in transition into water %	1.3 (4)	6.2 (4)	0.04*
FEV_1_/FVC decrease in transition into water %	5.3 (3)	2.2 (5)	0.17
MVV decrease in transition into water %	6.8 (4)	6.6 (5)	0.71
cMVV decrease in transition into water %	6.3 (3.2)	7.9 (3.2)	0.36

*Significant difference, #cMVV (35*FEV_1_)

### Ventilation during competition speed intensity swimming

The pulmonary minute ventilation (VE) during competition speed intensity swimming, which had the most respiratory symptoms triggered by different intensities, had a mean (SD) of 106 (4) lmin^1^ in females and 136 (14) lmin^1^ in males. The tidal volume had a mean (SD) of 2.5 (0.4) l in females and 3.6 (0.3) l in males. Breathing frequency (fb) had a mean (SD) of 56 (12) breathsmin^1^ in females and 54 (8) in males.

The minute ventilation (VE) during swimming was 72 (7)% in females and 75 (7)% in males of the calculated maximal voluntary ventilation (cMVV) on land and 76 (8)% in females and 80 (7)% in males of the cMVV measured in a water-oriented, face-immersed, prone swimming body position. The linear regression analysis showed that cMVV measured in water associated more (p<0.0001) with the VE during competition speed intensity swimming than the cMVV on land (p<0.001).

### Effect of competition speed swimming on spirometry findings

**Fig. 2:**
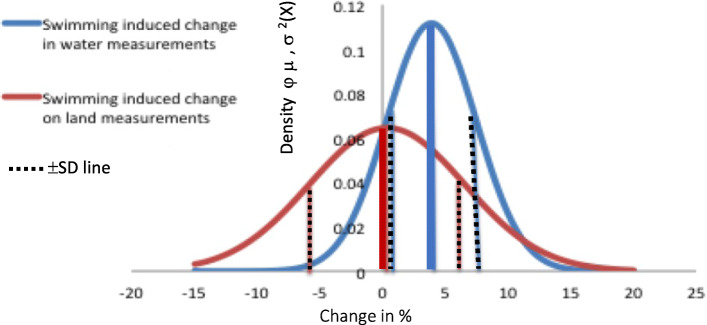
Normal distributions of the swimming-induced changes on FEV_1_ measured in water and on land. Wide vertical lines represent the mean change caused by swimming

Testing pulmonary functions on land and in water before and after swimming showed the effects of a water environment and swimming on pulmonary function. Swimming caused an increase in pulmonary function findings in water suggesting swimming-induced bronco-dilation. According to the results of this study, the mean differences between the change in spirometry values obtained before and after competition speed intensity swimming on land and in water are shown in the Fig.2.

Swimming was performed at competition speed intensity by 14 elite swimmers 7 females and 7 males.

## Discussion

### Study strategies used and the study limitations

The study strategy was set at aiming to provide evidence and to elucidate the swimming specific issues, which can affect the clinical testing quality in highly fit competitive swimmers.

This study had two main goals. The first goal was to elucidate the effects of water immersion on pulmonary function in highly fit swimmers and our second goal was to demonstrate evidence of the ventilation levels required to provoke a reaction in the airways of highly trained swimmers. With these goals this studys design strategy was to answer these goals by generating swimming-specific evidence and did not aim to imitate clinical testing.

A limitation of this study was that only healthy subjects were studied. Testing swimmers with asthma would have demanded further resources as more specialized personnel including physician specialized in sports and exercise medicine to be present in the testing. We thought to extend our study in the future testing swimmers with respiratory symptoms depending on the findings of this study. Another limitation was that, during swimming the water resistance during exhalation could not be achieved, as the testing tubes did not cause resistance.

### Effects of a water environment on the pulmonary function in competitive swimmers

The transition from land and a vertical sitting body position into a prone swimming body position in water caused a 18% decrease in spirometry variables, which may look minor. However, even a small effect of water immersion in water on pulmonary function may be essential, in swimmers with allergic sensitivity, pulmonary disease and a lowered the ratio of forced expiratory volume in one second (FEV_1_) and forced vital capacity (FVC); FEV_1_/FVC. That may be essential also especially with the extreme demands of competition speed swimming put upon ventilation function.

Water immersion showed gender differences in forced vital capacity FVC and the ratio of forced expiratory volume in one second (FEV_1_) and forced vital capacity (FVC); FEV_1_/FVC. The decrease in FVC was significantly greater in males by a mean of 6% in comparison to females with a mean of 1% (Table 2). Body composition with better buoyancy in female swimmers and smaller lungs may be the causes for the result .

A higher reporting of respiratory symptoms in asthmatic female swimmers in previous study[22]was presumably due to their smaller lungs compared to asthmatic males and FEV_1_/FVC change, which in turn, decreases more in females in transition from land into water. Furthermore, the opposite situation may occur. Larger lungs in male asthmatic swimmers with a minor decrease in FEV_1_/FVC in connection to water immersion connects to no reporting of respiratory symptoms during swimming [22].

The additional effect of a water environment may be a trigger for respiratory symptoms. For example, a previous study on swimmers baseline spirometry findings on land showed that a 4% decrease in FEV_1_/FVC associated significantly with the reporting of respiratory symptoms during swimming [23]. A similar association in endurance athletes was reported by Dickinson et al. [24].

### The measured effect of swimming on pulmonary function depends on the performed measurement protocol; on land or in water

The outcome of the effects of swimming on pulmonary function are different if measuring it on land or in water. Swimming caused an approximately 4% increase in forced expiratory volume in one second (FEV_1_) in water suggesting swimming-induced bronco-dilation. However, the findings measured on land had no difference in FEV_1_, which does not suggest any bronco-dilatation occurred (Fig.2). This finding may be due to water immersion-induced parasympathetic activation, which may affect pulmonary function. Pyhnen and Avela [25] demonstrated water immersion-induced parasympathetic neural activation. This may explain why the swimming-induced bronco-dilatation in the water is not observable, when measurements are performed after swimming on land.

It is notable that even though the mean swimming-induced change in FEV_1_ differed approximately 4% between land and water measurements, the SD`s overlapped (Fig.2). Thus, even though the differences between measurements on land and in water look minor at 18%, the difference of the spirometry results on land and in water was clear. Therefore, the results suggest a separate interpretation.

### Pulmonary ventilation during swimming at competition-speed intensity

During swimming when the intensity increases, the ventilation increases mainly by the VT, because the timing of breathing is dependent on the upper limb cycle. This is different from the exercise on land, where pulmonary ventilation increases in relation to the intensity of physical exercise on land during free breathing. During exercise the ventilation is increased first by increasing tidal volume (VT) and then by increasing the breathing frequency (fb) [26].

There were no sex differences in breathing frequency, but the VT was about one liter smaller in females. In both, in females and males, the VT was about half of the measured FVC.

The swimming-specific breathing pattern, which is restricted according to the upper limb cycle, causes swimmers to inhale their lungs fuller in comparison to breathing during physical exercise on land, and this may influence lung compliance and the work of breathing. However, the resistance of water during exhalation and hydrostatic pressure may compensate for that.

### Special observations for clinical testing in competitive swimmers

In clinical testing, a certain level of achieved pulmonary ventilation is used for triggering airway reactivity and respiratory symptoms. The target ventilation during clinical exercise challenge testing is 60% of the calculated maximal voluntary ventilation (cMVV) [12]. The questionnaire survey of 412 competitive swimmers showed that the competition speed intensity swimming triggered the most respiratory symptoms. The measurements in this study showed that the ventilation levels during that same intensity, were 80 and 76% of (cMVV). Thus, at the same intensity, the minute ventilation was approximately 1520% higher than is typically used as a target ventilation in clinical exercise challenge tests on land. This finding may be crucial, when respiratory symptoms in elite competitive swimmers are examined in clinical trials. Correspondingly, if highly fit competitive swimmer performs a challenge test on level of 60% of cMVV then that would be a ventilation level of zone II intensity swimming which is moderate intensity and triggers significantly less respiratory symptoms as shown in the Fig.1.

The exercise challenge test is typically performed on a treadmill or bicycle ergometer for 68min with a target heart rate of 8090% of predicted maximum of 220 minus the age, and ventilation should reach 4060% of the predicted cMVV (FEV_1_35). It is preferable to maintain the target ventilation for at least 4min during the challenge test [12]. However, the findings by Rundell et al. (2001)[27]and this study suggest that the higher exercise intensity near maximal effort may be required when elite athletes are tested.

In eucapnic voluntary hyperventilation (EVH) testing, the target ventilation is 85% of cMVV[28], which is closer to the ventilations observed during swimming at the competition speed.

Findings in a study by Pedersen et al.[29] are consistent with those found in this study. They studied different challenges such as EVH, a field-based exercise test by a swimming competition race, a laboratory-based exercise test and a methacholine test for competitive swimmers. They found that both EVH testing and a field-based exercise, a competition race challenge, showed post-challenge FEV_1_ fall the most positive for airway hyperresponsiveness [29].

Thus, the level of exercise intensity and ventilation during an exercise challenge may play an essential role. Carlsen et al.[30]demonstrated the importance of exercise load during an exercise challenge. In their study, 40% of the studied children had a diagnostic FEV_1_ fall of greater than 10%, when the exercise intensity was 85% of calculated maximum exercise load, but all 100% of the studied subjects had a positive finding when the exercise load was 95% of the calculated maxim [30]. That load would be considered close to the intensity swimming at competition speed.

Theoretically, a ventilation level of greater than 7580% of cMVV, which was obtained at very high intensity competition speed swimming, would be more appropriate for highly fit swimmers. That is consistent with the results for elite athletes in previous studies [24, 27].

Furthermore, Schwartz et al. [31] suggested that during exercise the ventilation rate and total ventilation strongly relate to the broncho-responsiveness in elite athletes.

The time duration, in their opinion, seemed unimportant [31].

In previous study during increased pulmonary ventilation, cooling and drying of airways, during increased pulmonary ventilation at high intensity physical exercise, are reported to be the stimuli for respiratory symptoms in previous studies [31,33] demonstrated an exercise-induced asthma (EIA) reaction, that is directly proportional to the thermal load on the airways and that the reaction is quantifiable in term of respiratory heat exchange (Deal et al. 1979). However, during swimming, the thermal loss is minimal, because the inhaled air is warm and humid. Therefore, swimming is considered beneficial for breathing.

Swimming as a low astmogenic sport induces fewer symptoms than running or cycling [34]. Therefore, especially testing during swimming in high fitness competitive swimmers, a higher intensity and more severe provocation, than typically used, may be required to trigger airway reactivity and symptoms.

Finally, the majority of the previous studies reports that exercise-challenge testing was performed without measuring the ventilation. However, the ventilation level may be crucial for the quality and to successfully perform the test. The findings of this study suggest, that observing the ventilation function could help to determine the desired exercise intensity for exercise-challenge tests especially for highly fit swimmers with developed pulmonary function [3, 35].

## Data Availability

Data are not available due to the datasets generated and analyzed during the current study not being publicly available due to the regulations of ethical committee statement but are available from the corresponding author on reasonable a request. However, it should be noted that the raw data availability is not possible to be provided outside the testing personnel of this study. This is ruled by the ethical committees of Helsinki and Uusimaa hospital district and University of Jyvskyl, which approved the testing protocol. Permission to deliver study data outside the study personnel requires new applications for the ethical committees and new approvals to deliver raw data outside the corresponding study personnel. Delivering the study data outside the testing personel may violate the anonymity of the study subjects and therefore the raw data availability is not recommended or allowed without additional applications to the ethical committees.

## Abbreviations

ATS: American Thoracic Society
BMI: Body mass index
ERS: European Respiratory Society
EVH: Eucapnic voluntary hyperventilation
FEV_1_: Forced expiratory volume in one second, liters (l)
FEV_1_/FVC: Ratio of forced expiratory volume in one second and forced vital capacity (%)
FINA: Fdration Internationale de Natation
FVC: Forced vital capacity, liters (l)
N: Number of subjects
n/N: Number of subjects concerned out of the whole population
OR: Odds ratio
PEF: Peak expiratory flow, liters per second (lsec^-1^)
MEF75: Maximum expiratory flow rate at 75% of vital capacity, liters per second (lsec^-1^)
MEF50: Maximum expiratory flow rate at 50% of vital capacity, liters per second, (lsec^-1^)
MVV: Maximal voluntary ventilation, liters per minute (lmin^-1^)
cMVV: Calculated maximal voluntary ventilation by 35xFEV_1_, liters per minute (lmin^-1^)
SD: Standard deviation
TLC: Total lung capacity, liters (lsec^-1^)
